# A Pilot MRI Study of Upward Gaze-Induced Intraocular Pressure Elevation in Thyroid Eye Disease

**DOI:** 10.3390/diagnostics16101521

**Published:** 2026-05-18

**Authors:** Muhammad Abumanhal, Chrisha Faye Habaluyas, Naomi Umezawa, Yasuhiro Takahashi

**Affiliations:** Department of Oculoplastic, Orbital & Lacrimal Surgery, Aichi Medical University Hospital, Nagakute 480-1195, Aichi, Japan; mabumanhal@hotmail.com (M.A.); chrishahab@gmail.com (C.F.H.); nume0828@yahoo.co.jp (N.U.)

**Keywords:** thyroid eye disease, intraocular pressure, upward gaze, magnetic resonance imaging

## Abstract

**Background/Objectives:** To investigate the scleral inferior rectus–optic nerve distance (SIROND) and its association with intraocular pressure (IOP) elevation during upward gaze in patients with thyroid eye disease (TED), based on magnetic resonance imaging (MRI) examinations. **Methods:** This prospective study included 20 eyes (13 patients) diagnosed with active TED. All patients underwent orbital MRI in both primary and upward gaze positions before and 6 months after steroid pulse therapy. The SIROND was measured on MRI. IOP was recorded in both gazes. Changes in SIROND, inferior rectus (IR) muscle volume, proptosis, optic disc and scleral morphology, and IOP were analyzed pre- and post-treatment. **Results:** SIROND significantly decreased from primary gaze to upward gaze both before and after treatment (*p* < 0.001). Following steroid pulse therapy, there were significant reductions in IR muscle volume and proptosis (*p* < 0.05). Correspondingly, SIROND significantly increased in both primary and more in upward gaze post-treatment (*p* < 0.05). Although IOP during upward gaze was significantly higher than that in primary gaze both before and after treatment (*p* < 0.001), the gaze-related difference in IOP (*p* = 0.059), as well as SIROND, tended to be smaller after treatment (*p* < 0.001). A larger reduction in gaze-related pre-treatment SIROND was associated with greater IOP elevation in upward gaze (*p* = 0.038). MRI showed no evidence of globe compression by the IR muscle, and optic disc morphology remained unchanged following treatment. **Conclusions:** SIROND may serve as supportive radiological evidence for IOP elevation induced by upward gaze in patients with TED.

## 1. Introduction

Thyroid eye disease (TED) is an autoimmune condition characterized by increased orbital tissue volume and subsequent proptosis, and anti-thyroid antibodies are thought to cross-react with antigens in orbital fibroblasts, triggering an inflammatory response [1]. This immune response leads to inflammatory cell infiltration in the extraocular muscles (EOMs) and orbital fat, resulting in EOM enlargement and increased orbital fat volume [1,2,3]. Orbital imaging is essential for diagnosing TED, as it clearly demonstrates these characteristic changes in orbital structures [4]. Among the available modalities, magnetic resonance imaging (MRI) is particularly valuable, as it can also assess disease activity, typically indicated by high signal intensity in EOMs and orbital fat [5].

Remodeling of orbital structures commonly results in elevated intraocular pressure (IOP) [6], which is often exacerbated during upward gaze [7,8,9,10,11,12,13]. This is primarily due to the frequent involvement and enlargement of the inferior rectus (IR) muscle in TED [14]. The thickened and fibrotic IR muscle becomes less elastic, creating mechanical resistance during upward gaze [15]. This resistance increases orbital congestion and episcleral venous pressures, contributing to IOP elevation [15,16]. Additionally, the stiffened IR muscle may exert direct compression on the globe during upward gaze, particularly near its insertion point [17].

Despite previous reports describing IOP elevation in upward gaze in TED [6,7,8,9,10,11,12,13,14,15,16,17,18,19], there remains a lack of objective radiological parameters that can quantitatively reflect the underlying orbital changes contributing to this phenomenon. Intraconal congestion and IR muscle enlargement are thought to play a key role, yet their relationship with IOP changes has not been well characterized using imaging-based metrics. Therefore, we introduce a novel radiological parameter, the scleral inferior rectus–optic nerve distance (SIROND), as a potential marker of infero-anterior intraconal crowding. We hypothesized that SIROND may reflect the degree of IR-related compression and congestion within the orbit and correlate with IOP elevation, particularly during upward gaze.

In this study, we evaluated and compared several radiographic parameters on MRI in both primary and upward gazes in patients with TED before and after steroid pulse therapy. These parameters were analyzed in relation to IOP elevation during upward gaze. To our knowledge, this is the first study to investigate IOP elevation during upward gaze in TED patients using MRI-based imaging analysis.

## 2. Materials and Methods

### 2.1. Study Design

**Figure 1:**
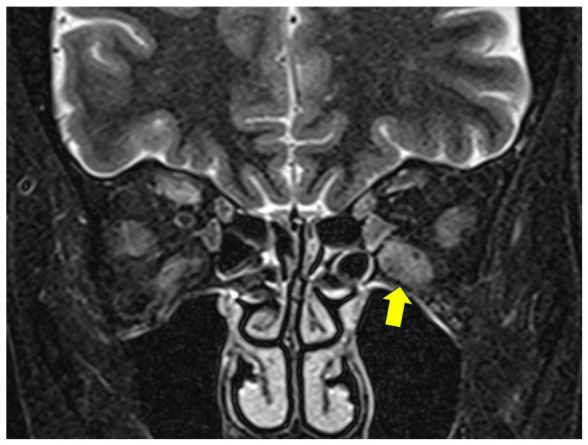
Inflammation of the left inferior rectus muscle shown as high signal intensity on a short tau inversion recovery (STIR) magnetic resonance image (arrow).

This prospective observational study included patients with TED who received steroid pulse therapy, with or without orbital irradiation, and demonstrated radiographic evidence of IR muscle inflammation on MRI from January 2024 to November 2024. This study was conducted in accordance with the STROBE (Strengthening the Reporting of Observational Studies in Epidemiology) guidelines for observational studies (Supplementary Material). The diagnosis of TED was established according to the criteria defined by Bartley [20]. The disease activity was determined by the presence of EOM inflammation on short tau inversion recovery (STIR) MRI, as a considerable number of Japanese patients with MRI-confirmed orbital soft tissue inflammation show a low clinical activity score (CAS) [21]. Active TED is characterized by increased water content within the EOMs and orbital soft tissues due to inflammatory infiltration and edema, which appears as high signal intensity on these sequences (Figure 1). MRI has been shown to provide an objective assessment of disease activity and may detect active inflammation even in cases with low CAS. This is particularly relevant in Japanese patients, among whom discordance between CAS and imaging findings has been reported [22,23,24].

#### 2.1.1. Inclusion Criteria

Patients were included if they had active, moderate-to-severe TED, received steroid pulse therapy with or without orbital irradiation, and demonstrated radiographic evidence of IR muscle inflammation on STIR MRI.

#### 2.1.2. Exclusion Criteria

Patients were excluded if they had a history of orbital surgery, strabismus surgery, or glaucoma surgery, or if they were using anti-glaucomatous eyedrops. Eyes without IR muscle inflammation were also excluded.

#### 2.1.3. Study Workflow

After meeting the inclusion criteria, patients underwent (1) baseline clinical and MRI assessment in both primary and upward gaze positions; (2) steroid pulse therapy; (3) post-treatment clinical and MRI assessment at 6 months using the same protocol; and (4) radiological and statistical analyses.

### 2.2. Data Collection

The following data were collected: age, sex, laterality, thyroid disease (Graves’ disease or Hashimoto’s thyroiditis), and thyroid functional status.

Ophthalmic evaluation included IOP, optic disc morphology, and proptosis, assessed both before and 6 months after steroid pulse therapy. IOP was measured using a rebound tonometer (iCARE IC200; Icare Finland, Vantaa, Finland) in the primary gaze and 20° upward gaze [13]. Optic disc morphology was evaluated using optical coherence tomography (OCT) (CIRRUS HD-OCT; Carl Zeiss Meditec Co., Ltd., Tokyo, Japan), with parameters including mean retinal nerve fiber layer (RNFL) thickness and vertical and horizontal cup/disc (C/D) ratios. The grade of proptosis was measured using a Hertel exophthalmometer. Clinical assessment was performed by the same experienced examiner (YT) before and six months after steroid pulse therapy using a standardized protocol.

### 2.3. MRI

MRI was performed using a 1.5-Tesla scanner (Magnetom Abant™; Siemens Healthcare, Erlangen, Germany), with the patients in the supine position both before and 6 months after steroid pulse therapy. T1- and T2-weighted gradient-echo sequences were acquired (T1—repetition time: 500 ms, echo time: 10 ms, field of view: 140 × 140 mm, matrix: 256 × 220, section thickness: 2.5–3 mm with a 0.6 mm gap between slices; T2—repetition time: 4000 ms, echo time: 100 ms, all other parameters were the same as in T1).

**Figure 2:**
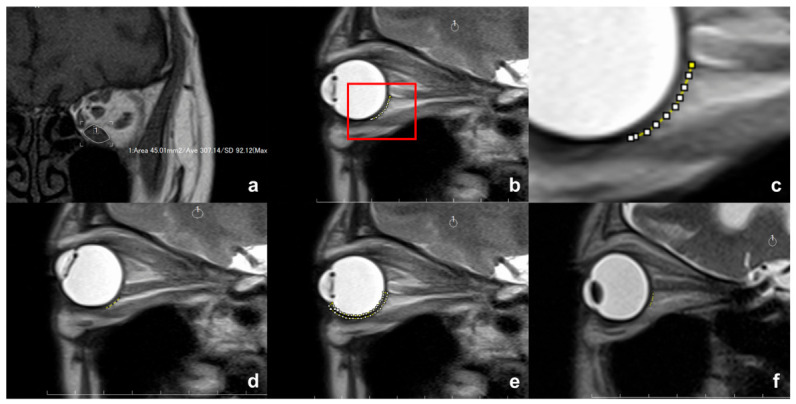
(**a**) Measurement of the cross-sectional area of the inferior rectus (IR) muscle. (**b**–**d**) Measurement of scleral inferior rectus–optic nerve distance (SIROND) in the primary gaze (**b**); (**c**) magnified image of the rectangular area in part (**b**) and upward gaze (**d**) (yellow broken lines). (**e**) Measurement of the cross-sectional area of the sclera from the iridocorneal angle to the inferior margin of the optic nerve at its insertion into the globe (yellow broken line). (**f**) A shorter line of the non-contact plane (yellow broken line) with enlargement of the anterior portion of the IR muscle.

The cross-sectional areas of the IR muscle were measured on a coronal T1-weighted MRI in each slice where the EOM was visualized using the measuring tool available in the MRI viewer (ShadeQuest/ViewR™; Yokogawa Medical Solutions Corporation, Tokyo, Japan) (Figure 2a). The IR muscle volume was calculated as the total summed of cross-sectional areas multiplied by slice thickness.

T2-weighted sagittal section of cine-mode MRI through the IR muscle and optic nerve (ON) was taken in a similar fashion as in a previous study [25]. Patients were instructed to fixate on a vertical row of numbered marks placed on the inner aspect of the scanner bore above their heads. The vertical row consisted of 5 fixation marks placed at intervals corresponding to a 10° gaze angle difference (from −20° to 20°). The slices obtained in the primary and 20° upward gazes were used for analyses. In patients with severe IR muscle enlargement and hypotropia, the eye with milder restriction of upward gaze was fixed to the fixation marks. Due to the obscured insertion of the IR muscle into the globe, the length of the contact plane between the globe and the IR muscle could not be directly measured. Instead, the non-contact distance from the inferior margin of the ON at its insertion into the globe to the posterior point of the IR muscle insertion was measured along the scleral surface and referred as the scleral IR-ON distance (SIROND) (Figure 2b,c). A shorter length of SIROND indicates greater contact between the globe and IR muscle, resulting in more compression of the globe by the IR muscle. The number of patients in whom the sclera was yielded by compression of the IR muscle was recorded. Since the cornea was obscure on MRI, the cross-sectional area of the sclera was measured from the level of iridocorneal angle to the inferior margin of the ON at its insertion into the globe (Figure 2d). Those two measurements were performed by one of the authors (YT) using ImageJ 1.54g software (National Institute of Health, Bethesda, MD, USA).

All MRI measurements, including SIROND, cross-sectional area, and muscle volume, were performed by a single experienced examiner (YT) using a standardized protocol to ensure consistency.

### 2.4. Treatment

Patients underwent three cycles of steroid pulse therapy under a regimen of body weight × 10 mg/kg/day of methylprednisolone for 3 consecutive days per cycle, based on the guideline for treatment of thyroid eye disease established by the Japanese Committee for Diagnostic Criteria and Guideline to Medical Care for Thyroid-Associated Malignant Exophthalmos [26]. The total cumulative dose of methylprednisolone did not exceed 8 g in all patients. Regarding orbital irradiation, a total dose of 20 Gy in 10 fractions was delivered during hospitalization for steroid pulse therapy.

### 2.5. Statistical Analyses

Patient data and measurement results were expressed as means ± standard deviations (SDs). Decreasing rates of SIROND were calculated as follows:

$$\frac{\left(SIROND\;measured\;in\;the\;primary\;position\right)-(SIROND\;measured\;in\;the\;upward\;gaze)}{SIROND\;measured\;in\;the\;primary\;gaze}\;\times \;100$$

The normal distribution of measurement results was examined using the Shapiro–Wilk test, and dependent continuous variables were compared using the paired *t*-test or Wilcoxon signed-rank test, based on the results of the Shapiro–Wilk test. The Mann–Whitney-U test was used for comparison of independent continuous variables. The relationship between changes in pre-treatment IOP by gazes and clinical and radiographic variables using univariate and multivariate linear regression analyses. The variables included the Hertel exophthalmometric value, IR muscle volume, pre-treatment SIROND in the primary position, difference in the pre-treatment SIROND between gazes, and decreasing rate of the pre-treatment SIROND. All statistical analyses were performed using SPSS™ version 26 software (IBM Japan, Tokyo, Japan). Two-tailed *p* values < 0.05 were deemed to indicate statistical significance.

## 3. Results

Data on patient characteristics are shown in Table 1. This study included 20 eyes from 13 patients (2 males and 11 females; mean age, 55.3 ± 17.4 years). Bilateral inflammatory enlargement of the IR muscle was observed in 7 patients, and 6 eyes without IR muscle inflammation were excluded. All patients were diagnosed with Graves’ disease, and thyroid status was controlled to euthyroid in 9 of them. Orbital irradiation was performed in 9 patients, while the remaining 4 patients did not undergo it due to either young age (2 patients) or pre-existing diabetes mellitus (2 patients). EOM involvement other than the IR muscle was observed in 18 eyes.

Measurement results are presented in Table 2. Before treatment, IOP in the primary and 20° upward gazes was 15.0 ± 4.0 and 20.5 ± 6.4 mmHg, respectively. After steroid pulse therapy, IOP decreased to 13.9 ± 2.5 mmHg in the primary gaze and 17.2 ± 4.6 mmHg in the upward gaze. IOP in the upward gaze was significantly higher during both pre- and post-treatment periods (both, *p* < 0.001). Although the reduction in IOP in the primary gaze after treatment did not reach statistical significance (*p* = 0.167), IOP in upward gaze showed a significant decrease between pre- and post-treatment periods (*p* = 0.012). In addition, 13 of 20 eyes showed reduction in IOP in both gaze positions. The difference in IOP between the two gaze positions was 5.5 ± 4.8 mmHg before treatment and 3.3 ± 3.9 mmHg after steroid pulse therapy, but this difference did not reach statistical significance (*p* = 0.059). The mean RNFL thickness and vertical and horizontal C/D ratios did not significantly change after steroid pulse therapy (*p* > 0.050). Hertel exophthalmometric values significantly decreased from 17.4 ± 3.2 mm to 16.6 ± 2.7 mm (*p* = 0.003).

The IR muscle volume significantly decreased from 1464.1 ± 458.9 mm^3^ before steroid pulse therapy to 1219.6 ± 363.6 mm^3^ after treatment (*p* < 0.001). Before treatment, the SIROND significantly decreased from 1.01 ± 0.42 mm in the primary gaze to 0.63 ± 0.35 mm in the upward gaze (*p* < 0.001). After steroid pulse therapy, the SIROND significantly increased in both gaze positions (primary gaze, 1.18 ± 0.29 mm, *p* = 0.020; upward gaze, 0.94 ± 0.32 mm, *p* < 0.001). Despite this increase, the SIROND remained significantly shorter in the upward gaze compared to the primary gaze after treatment (*p* < 0.001). However, the reduction rate of SIROND between the two gaze positions was significantly smaller following steroid pulse therapy (pre-treatment, 40.0 ± 15.2% vs. post-treatment, 22.6 ± 14.0%; *p* < 0.001). None of the eyes showed that the sclera yielded by compression of the IR muscle, as depicted on MRI. Consequently, the cross-sectional area of the sclera was not significantly different between pre- and post-treatment periods in the primary and upward gazes, as well as between the primary and upward gazes in both pre- and posttreatment periods (all, *p* > 0.050).

Eyes with a shorter SIROND likely due to enlargement of the anterior portion of the IR muscle seemed to exhibit higher IOP elevation in upward gaze (Figure 2e). We, therefore, classified eyes into two groups: those with IOP elevation of ≥5 mmHg (*n* = 11) and those with IOP elevation of <5 mmHg (*n* = 9). Eyes in the ≥5 mmHg group showed a significantly larger reduction rate of SIROND between the two gaze positions (46.4 ± 12.0% vs. 32.2 ± 15.7%, *p* = 0.038). However, pre-treatment SIROND values were not significantly different between the groups in both the primary gaze (0.96 ± 0.44 mm vs. 1.08 ± 0.41 mm, *p* = 0.766) and upward gaze (0.53 ± 0.31 mm vs. 0.75 ± 0.37 mm, *p* = 0.175). Since univariate linear regression analyses did not identify any variables as a significant predictor of upward-gaze-induced IOP (all, *p* > 0.050) (Table 3), multiple linear regression analysis was not performed.

## 4. Discussion

In this study, we introduced SIROND for the first time as a radiological marker that may serve as an indicator of IOP elevation in TED. The dynamic change in this distance between primary and upward gaze could help explain the mechanism behind IOP elevation during upward gaze.

While previous studies have demonstrated IOP elevation in upward gaze among TED patients [6,7,8,9,10,11,12,13,14,15,16,17,18,19], our study also confirmed a significant increase in IOP during upward gaze both before and after steroid pulse therapy. Correspondingly, we observed that the radiological marker SIROND significantly decreased from the primary to upward gaze in both pre- and post-treatment assessments. We also found that, in pre-treatment measurements, patients with shorter SIROND in the primary gaze exhibited greater IOP elevation in upward gaze. Following treatment, the reduction in SIROND between primary and upward gaze was less pronounced, and, correspondingly, the difference in IOP between the two gaze positions was reduced. These findings may indicate that direct compression of the globe and episcleral veins by an enlarged IR muscle raises IOP and serve as “radiological evidence” supporting the mechanism of IOP elevation during upward gaze.

SIROND may also serve as a radiological marker of intraconal congestion in TED. We hypothesized that greater IR enlargement and intraconal crowding would result in a shorter SIROND, due to the muscle’s extended insertion along the globe. We found that following steroid pulse therapy, which reduced inflammation and congestion, SIROND significantly increased in both primary and upward gazes. This was supported by concurrent significant improvement in proptosis and reduction in IR muscle volume. Additionally, in upward gaze, the thickened and fibrotic IR muscle imposes mechanical resistance and elevates intraconal pressure, resulting in consistently shorter SIROND compared to the primary gaze, both before and after treatment. Clinically, these findings suggest that SIROND may provide a quantitative and objective non-invasive imaging biomarker for assessing intraconal congestion, monitoring treatment response, and potentially identifying patients at risk for gaze-related IOP elevation. This may be particularly valuable in cases where conventional clinical parameters are insufficient to fully capture disease activity or pressure-related changes.

MRI showed no evidence of scleral deformation due to IR muscle compression in any eye. This was further supported by the unchanged cross-sectional area of the sclera between primary and upward gazes, as well as before and after treatment. One possible explanation for these findings is that IOP was sufficiently elevated to prevent scleral deformation despite direct compression of the globe by the enlarged IR muscle. Alternatively, these results suggest that direct globe compression by the IR muscle, as proposed in previous studies [13,17], is less likely the primary mechanism of IOP elevation in upward gaze. Instead, elevated orbital tissue volume and intraconal congestion are likely to raise retrobulbar and episcleral venous pressures [13,17,27], contributing to IOP elevation. Additionally, mucopolysaccharide accumulation in the trabecular meshwork may impair aqueous humor outflow, further increasing IOP [27].

Monitoring and managing IOP in patients with TED are crucial. In our study, although a trend toward IOP reduction was observed following steroid pulse therapy, optic disc morphology, such as vertical and horizontal C/D ratios, and structural parameters including RNFL thickness remained unchanged. This supports the understanding that optic nerve damage due to elevated IOP is often irreversible [28]. Therefore, careful and proactive monitoring of IOP in TED patients is strongly recommended.

Although IOP values remained within the normal range, a significant difference was observed between primary and upward gaze, indicating a measurable effect of intraconal congestion on IOP in TED. The absence of markedly elevated IOP values may be attributed to the limited sample size and the inclusion of fewer patients with severe disease. In addition, we could not find a significant association between upward-gaze-induced IOP and SIROND in the univariate linear regression analyses. This may be attributed to the large SD in IOP changes between the gaze positions, indicating a considerable data dispersion within the study sample. Despite this limitation, the consistent trend supports SIROND as a potentially sensitive imaging marker for detecting subtle pressure-related changes, even when IOP does not exceed normal limits.

Patients received systemic steroid treatment with or without orbital irradiation, as teprotumumab treatment was not available in Japan at the time this study started. To date, no studies have reported changes in upward-gaze-induced IOP and SIROND on MRI post-teprotumumab treatment. However, previous studies have shown that teprotumumab significantly reduces IOP in the primary gaze and decreases EOM volume [29,30,31,32,33]. Additionally, teprotumumab has demonstrated greater improvement in extraocular motility, compared to systemic steroid [1,34,35], suggesting it may lead to a more substantial reduction in IR muscle volume, SIROND, and IOP during upward gaze in comparison to steroid therapy.

This study has several limitations. The sample size was relatively small, which may limit the generalizability of the findings. While IOP measurements were conducted in the upright back position, the MRI examinations were performed in the supine position. This positional difference could have introduced variability in the correlation between anatomical and pressure-related findings [36]. The lack of a control group of healthy participants limits our ability to determine whether the observed radiological changes are specific to TED. Future studies with larger cohorts and appropriate control groups are warranted to validate our findings and further establish the clinical utility of SIROND as a radiological marker.

## 5. Conclusions

This study suggests the potential utility of SIROND as a novel radiological marker of intraconal congestion in TED and as supportive imaging evidence for upward gaze induced IOP elevation in affected patients. Radiological signs of globe compression were not observed, which may indicate that it is less likely to be a major contributing factor to IOP elevation in this context. Still, further studies are needed to confirm these findings.

## Figures and Tables

**Table 1:** Data on patient characteristics and measurement results.

Number of patients/eyes	13/20
Sex (male/female)	2/11
Side (right/left)	8/12
Age (years)	55.3 ± 17.4
Number of patients with orbital irradiation	9
Number of eyes with involvement of the extraocular muscles other than the inferior rectus muscle (overlapped)	
Superior rectus/levator palpebrae superioris muscles	13
Medial rectus muscle	9
Lateral rectus muscle	13
Superior oblique muscle	6
Inferior oblique muscle	5

**Table 2:** Pre- and post-treatment measurement results with statistical comparison.

	Pre-Treatment	Post-Treatment	95% CI	Size Effect	*p* Value (Pre vs. Post)
IOP (mmHg)					
Primary position	15.0 ± 4.0	13.9 ± 2.5		−0.309	0.167 *
Upward gaze	20.5 ± 6.4	17.2 ± 4.6		−0.559	0.012 *
95% CI		−4.931 to −1.759			
Size effect	−0.852	3.531			
*p* value (primary vs. upward)	<0.001 *	<0.001 ^†^			
Mean RNFL thickness (µm)	91.5 ± 10.2	92.6 ± 9.2	−3.467 to 1.267	5.268	0.343 ^†^
Vertical C/D ratio	0.49 ± 0.19	0.47 ± 0.19	−0.024 to 0.071	0.105	0.310 ^†^
Horizontal C/D ratio	0.52 ± 0.18	0.50 ± 0.18	−0.022 to 0.054	0.085	0.393 ^†^
Hertel exophthalmometric value (mm)	17.4 ± 3.2	16.6 ± 2.7	0.326 to 1.324	1.112	0.003 ^†^
IR muscle volume (mm^3^)	1464.1 ± 458.9	1219.6 ± 363.6	133.105 to 355.895	247.956	<0.001 ^†^
SIROND (mm)					
Primary position	1.01 ± 0.42	1.18 ± 0.29	−0.311 to −0.029	0.313	0.020 ^†^
Upward gaze	0.63 ± 0.35	0.94 ± 0.32	−0.411 to −0.194	0.241	<0.001 ^†^
95% CI	0.228 to 0.440	0.193 to 0.323			
Size effect	0.223	0.137			
*p* value (primary vs. upward)	<0.001 ^†^	<0.001 ^†^			
SIROND reduction rate between primary and upward gaze (%)	40.0 ± 15.2	22.6 ± 14.0	10.000 to 23.848	0.165	<0.001 ^†^
Cross-sectional area of sclera (mm^2^)					
Primary position	60.40 ± 13.89	57.59 ± 13.57	−4.703 to 10.323	16.723	0.443 ^†^
Upward gaze	61.08 ± 17.37	59.52 ± 12.23	−6.092 to 9.213	17.034	0.674 ^†^
95% CI	−5.535 to 3.198	−3.516 to 0.794			
Size effect	9.194	4.537			
*p* value (primary vs. upward)	0.726 ^†^	0.144 ^†^			

CI, confident interval; IOP, intraocular pressure; RNFL, retinal nerve fiber layer; C/D, cup/disc; IR, inferior rectus; SIROND, scleral inferior rectus–optic nerve distance. * Wilcoxon signed-rank test. ^†^ Paired *t*-test.

**Table 3:** Results of the univariate regression analyses.

Dependent Variables	Univariate	
	Crude Coefficient (95% CI)	*p* Value
Hertel exophthalmometric value	−0.130 (−0.859 to 0.599)	0.712
IR muscle volume	0.002 (−0.003 to 0.007)	0.498
SIROND		
Primary position	0.160 (−5.410 to 5.730)	0.953
Gaze-related difference	2.709 (−9.432 to 14.850)	0.645
Gaze-related reduction rate	3.919 (−11.451 to 19.289)	0.599

IR, inferior rectus; SIROND, scleral inferior rectus–optic nerve distance; CI, confidence interval.

## Data Availability

The data are not publicly available due to ethical reasons. But, the datasets generated during and/or analyzed during this study can be made available by the corresponding author upon reasonable request.

## Supplementary Materials

The following supporting information can be downloaded at: https://www.mdpi.com/article/10.3390/diagnostics16101521/s1, File S1: STROBE Statement—checklist of items that should be included in reports of observational studies.

## Abbreviations

The following abbreviations are used in this manuscript:

TED	Thyroid eye disease
EOM	Extraocular muscle
MRI	Magnetic resonance imaging
IOP	Intraocular pressure
IR	Inferior rectus
OCT	Optical coherence tomography
RNFL	Retinal nerve fiber layer
C/D	Cup/disc
ON	Optic nerve
SIROND	Scleral inferior rectus–optic nerve distance
SD	Standard deviation
IRB	Institutional review board

## References

[1] Hiromatsu Y., Ishikawa E., Kozaki A., Takahashi Y., Tanabe M., Hayashi K., Imagawa Y., Kaneda K., Mimura M., Dai X. (2025). A randomised, double-masked, placebo-controlled trial evaluating the efficacy and safety of teprotumumab for active thyroid eye disease in Japanese patients. Lancet Reg. Health West. Pac..

[2] Menconi F., Marcocci C., Marinò M. (2014). Diagnosis and classification of Graves’ disease. Autoimmun. Rev..

[3] Meyer P., Das T., Ghadiri N., Murthy R., Theodoropoulou S. (2019). Clinical pathophysiology of thyroid eye disease: The Cone Model. Eye.

[4] Luccas R., Riguetto C.M., Alves M., Zantut-Wittmann D.E., Reis F. (2024). Computed tomography and magnetic resonance imaging approaches to Graves’ ophthalmopathy: A narrative review. Front. Endocrinol..

[5] Song Y., Li T., Tang W., Lv Q., Zhang X.X., Zhou W.Y., Shi Y.Q. (2025). Application progress of magnetic resonance imaging in thyroid associated ophthalmopathy. Front. Endocrinol..

[6] Cockerham K.P., Pal C., Jani B., Wolter A., Kennerdell J.S. (1997). The prevalence and implications of ocular hypertension and glaucoma in thyroid-associated orbitopathy. Ophthalmology.

[7] Spierer A., Eisenstein Z. (1991). The role of increased intraocular pressure on upgaze in the assessment of Graves ophthalmopathy. Ophthalmology.

[8] Girod D.A., Orcutt J.A., Cummings C.W. (1993). Orbital decompression for preservation of vision in Graves’ ophthalmopathy. Arch. Otolaryngol. Head. Neck Surg..

[9] Ben Simon G.J., Syed H., Douglas R., Schwartz R., Goldberg R.A., McCann J.D. (2006). Clinical manifestations and treatment outcome of optic neuropathy in thyroid-related orbitopathy. Ophthalmic Surg. Lasers Imaging.

[10] Danesh-Meyer H.V., Savino P.J., Deramo V., Sergott R.C., Smith A.F. (2001). Intraocular pressure changes after treatment for Graves’ orbitopathy. Ophthalmology.

[11] Onaran Z., Konuk O., Sö O., Yücel C., Ünal M. (2014). Intraocular pressure lowering effect of orbital decompression is related to increased venous outflow in Graves orbitopathy. Curr. Eye Res..

[12] Norris J.H., Ross J.J., Kazim M., Selva D., Malhotra R. (2012). The effect of orbital decompression surgery on refraction and intraocular pressure in patients with thyroid orbitopathy. Eye.

[13] Takahashi Y., Nakamura Y., Ichinose A., Kakizaki H. (2014). Intraocular pressure change with eye positions before and after orbital decompression for thyroid eye disease. Ophthalmic Plast. Reconstr. Surg..

[14] Gonçalves A.C., Gebrim E.M., Monteiro M.L. (2012). Imaging studies for diagnosing Graves’ orbitopathy and dysthyroid optic neuropathy. Clinics.

[15] Li X., Bai X., Liu Z., Cheng M., Li J., Tan N., Yuan H. (2021). The effect of inferior rectus muscle thickening on intraocular pressure in thyroid-associated ophthalmopathy. J. Ophthalmol..

[16] Nassr M.A., Morris C.L., Netland P.A., Karcioglu Z.A. (2009). Intraocular pressure change in orbital disease. Surv. Ophthalmol..

[17] Takahashi Y., Vaidya A. (2023). Secondary effects of orbital decompression in thyroid eye disease: A review. Semin. Ophthalmol..

[18] Hsia Y., Wei Y.H., Liao S.L. (2023). The changes in ocular biomechanical response parameters and intraocular pressure after surgical treatment for thyroid eye disease. Investig. Ophthalmol. Vis. Sci..

[19] Kim J.W., Ko J., Woo Y.J., Bae H.W., Yoon J.S. (2018). Prevalence of ocular hypertension and glaucoma as well as associated factors in Graves’ orbitopathy. J. Glaucoma.

[20] Bartley G.B., Gorman C.A. (1995). Diagnostic criteria for Graves’ ophthalmopathy. Am. J. Ophthalmol..

[21] Takahashi Y., Vaidya A., Kakizaki H. (2023). Risk factors for development of superior limbic keratoconjunctivitis in thyroid eye disease in Japanese. Graefes Arch. Clin. Exp. Ophthalmol..

[22] Gou X., Cheng J., Li T., Zhao W., Wang Y., Zhang X., Hong N. (2026). Research progress and clinical utility of multi-parameter orbital MRI in thyroid-associated ophthalmopathy. Eur. J. Radiol..

[23] Zhang H., Lu T., Liu Y., Jiang M., Wang Y., Song X., Fan X., Zhou H. (2024). Application of Quantitative MRI in Thyroid Eye Disease: Imaging Techniques and Clinical Practices. J. Magn. Reson. Imaging..

[24] Cai M., Yang J., Li X., Hu Y., Liao H., Xiong C. (2025). MRI-based SIR quantitative biomarkers: A novel imaging diagnostic strategy for thyroid eye disease activity staging. Front. Endocrinol..

[25] Kakizaki H., Selva D., Leibovitch I. (2010). Dynamic study of the medial and lateral recti capsulopalpebral fasciae using cine mode magnetic resonance imaging. Ophthalmology.

[26] (2018). The Japanese Committee for Diagnostic Criteria and Guideline to Medical Care for Thyroid-Associated Malignant Exophthalmos. The Guideline for Treatment of Thyroid Eye Disease. http://www.japanthyroid.jp/doctor/img/basedou02.pdf.

[27] Karhanová M., Kalitová J., Kovář R., Schovánek J., Karásek D., Čivrný J., Hübnerová P., Mlčák P., Šín M. (2022). Ocular hypertension in patients with active thyroid-associated orbitopathy: A predictor of disease severity, particularly of extraocular muscle enlargement. Graefes Arch. Clin. Exp. Ophthalmol..

[28] Diekmann H., Fischer D. (2013). Glaucoma and optic nerve repair. Cell Tissue Res..

[29] Adetunji M.O., Nguyen B.J., McGeehan B., Tamhankar M.A., Briceño C.A. (2022). Effect of teprotumumab on intraocular pressure in thyroid-associated ophthalmopathy. Taiwan J. Ophthalmol..

[30] Jain A.P., Gellada N., Ugradar S., Kumar A., Kahaly G., Douglas R. (2022). Teprotumumab reduces extraocular muscle and orbital fat volume in thyroid eye disease. Br. J. Ophthalmol..

[31] Ugradar S., Parunakian E., Zimmerman E., Malkhasyan E., Raika P., Douglas R.N., Kossler A.L., Douglas R.S. (2025). Clinical and radiologic predictors of response to teprutoumumab: A 3D volumetric analysis of 35 patients. Ophthalmic Plast. Reconstr. Surg..

[32] Richkind H., Shoji M.K., Walker E., Routzong M.R., Kikkawa D.O., Granet D., Rudell J. (2026). Differential change in extraocular muscle volume after teprotumumab for thyroid eye disease. Orbit.

[33] Kozaki A., Takahashi Y., Tajiri Y., Wang Z., Hayashida T., Kumar A., Hiromatsu Y., Mimura M. (2026). Reduction in extraocular muscle and orbital fat volumes after teprotumumab treatment in Japanese patients with thyroid eye disease: An exploratory magnetic resonance imaging analysis from the OPTIC-J trial. Jpn. J. Ophthalmol..

[34] Douglas R.S., Dailey R., Subramanian P.S., Barbesino G., Ugradar S., Batten R., Qadeer R.A., Cameron C. (2022). Proptosis and diplopia response with teprotumumab and placebo vs the recommended treatment regimen with intravenous methylprednisolone in moderate to severe thyroid eye disease: A meta-analysis and matching-adjusted indirect comparison. JAMA Ophthalmol..

[35] Lustig-Barzelay Y., Yagoda D., Zunz E., Hamed-Azzam S., Avisar I., Kehat-Ophir S., Gur Z., Cukierman-Yaffe T., Agmon-Levin N., Landau-Prat D. (2025). Time to improvement following teprotumumab treatment of thyroid eye disease: Real world experience. Graefes Arch. Clin. Exp. Ophthalmol..

[36] Jorge J., Ramoa-Marques R., Lourenço A., Silva S., Nascimento S., Queirós A., Gonzalez-Méijome J.M. (2010). IOP variations in the sitting and supine positions. J. Glaucoma.

